# Enhancing QoE for Mobile Users by Environment-Aware HTTP Adaptive Streaming

**DOI:** 10.3390/s18113645

**Published:** 2018-10-27

**Authors:** Weizhan Zhang, Hao He, Shuyan Ye, Zhiwen Wang, Qinghua Zheng

**Affiliations:** MOEKLINNS Lab, School of Electronics and Information Engineering, Xi’an Jiaotong University, Xi’an 710049, China; hehao7@stu.xjtu.edu.cn (H.H.); ysy616291@stu.xjtu.edu.cn (S.Y.); wzw@xjtu.edu.cn (Z.W.); qhzheng@xjtu.edu.cn (Q.Z.)

**Keywords:** sensors in mobile phone, environment-aware, HTTP adaptive streaming, quality of experience

## Abstract

HTTP adaptive streaming (HAS) has become a dominated media streaming paradigm in today’s Internet, which enriches the user’s experience by matching the video quality with the dynamic network conditions. A range of HAS mechanisms have been proposed to enhance the Quality of Experience (QoE). However, existing mechanisms ignore the environmental impact in the QoE evaluation of mobile users, while the popularity of mobile video allows users to watch videos in diversified scenarios. In this paper, we propose an environment-aware HAS scheme that fully concentrates on the different criteria for evaluating video QoE under different environments. Using the advantage of the sensors in mobile phones, the scheme constructs and validates a video QoE model based on environment perception and then designs a model-driven, environment-aware HAS rate adaptation algorithm. We also evaluate the scheme with an environment-aware DASH (Dynamic Adaptive Streaming over HTTP) player in real mobile environments. Compared to the benchmark HAS mechanism, the experimental results demonstrate that our scheme can provide appropriate differentiated rate adaptation for different environments, resulting in a higher QoE.

## 1. Introduction

Nowadays, with the development of mobile Internet technology and multimedia applications, video streaming is becoming one of the most dominant applications in the mobile Internet. However, unpredictable variation in the mobile network in terms of bandwidth, delay jitter, packet loss, etc., causes an unsatisfactory Quality of Experience (QoE) for mobile users, due to, for example, video stalling or low clarity. Therefore, an increasing number of video applications are employing HAS (HTTP Adaptive Streaming) to cope with the unpredictable changes in the mobile network. In HAS, one video stream is divided into multiple segments, and each segment is encoded into different bit rate levels. The mobile client can request an appropriate bit rate segment for the next part of the video according to the bandwidth or buffer available, and thus, provide adaptation to the changing network and server conditions. Due to the dynamic adaptability and the strong penetrability of HTTP, recently, the use of HAS technology has become widespread in mobile video streaming services. Existing famous standards and protocols include Apple’s HTTP Live Streaming (HLS), Adobe’s HTTP Dynamic Streaming (HDS), Microsoft’s HTTP Smooth Streaming (HSS), MPEG’s Dynamic Adaptive Streaming over HTTP (DASH), and the native HAS implementation by HTML5.

Besides the abovementioned standardized solutions, the provision of an optimal viewing experience to the end-users of HAS has been intensively studied by researchers [1]. Researchers have usually concentrated on reducing bit rate switches and interruptions to video playback, and providing higher bandwidth utilization, most of which result in a higher QoE. Therefore, the studies of QoE evaluation methodologies and performance metrics play a key role in optimizing the delivery of HAS services [2,3]. Some important QoE evaluating metrics of HAS have been well studied, such as initial delay, stallings, the bit rate switching frequency by video adaptation strategy, and video quality with different resolutions or quantizations.

However, since QoE is the subjective perception of the quality and performance of the application, the QoE evaluating metrics of HAS may play different roles under different environmental scenarios. In particular, mobile end users of HAS are typically present in different environments, such as in a library or on a bus. Under these varying environments, QoE metrics impose different influences on the user’s viewing experience. For example, in a noisy and moving bus, the fluency of videos is generally what mobile users focus on. On the other hand, in a static and quiet library, HAS video viewers may pay more attention to the quality of videos. As a result, stallings and video quality will have different weights on the QoE to end users in different environments. Ignoring the differences of the environment may greatly reduce the accuracy of the corresponding QoE models.

Therefore, in this paper, we propose an environment-aware QoE model and the corresponding model-driven HAS rate adaptation algorithm, which have been implemented by an environment-aware DASH player in real mobile scenarios. Firstly, the environments are classified by the sensor data from the mobile client. Then, the environment-aware QoE model is established by considering the different effects of stalling and quality in different types of environments on QoE. The QoE model is verified by subjective scores from volunteers in the different environments. Finally, an environment-aware HAS rate adaptation algorithm is proposed based on the established QoE model. The algorithm is upgraded from a newly introduced HAS algorithm, BOLA [4]. Notice that the scheme puts forward a generalized idea that the environmental scenarios should be contained in the HAS scheme which could be adopted by other existing HAS solutions. From the objective and subjective evaluation results, it is demonstrated that the scheme can provide a strategy with an appropriate differentiated rate according to different scenarios, resulting in the delivery of an enhanced QoE.

The main contributions of this paper are summarized as follows:

- The proposed scheme targets the provision of differentiated HAS services under different environments and the improvement of the QoE associated with HAS for mobile users. The scheme carefully studies how the different QoE evaluation metrics enhance the QoE of HAS, and tries to provide a generalized environment-aware QoE model for HAS that extends the domain of the traditional QoE modeling methodology.
- The proposed HAS rate adaptation algorithm takes the environment-aware QoE model into account and provides a synergistic solution. The algorithm is carried out with the modification of the benchmark HAS client as a case study. The local rate adaptation decisions of each client are modified by the environment-aware strategy. In this manner, through a well-designed HAS rate adaptation strategy with the environment perception, the algorithm can provide appropriate differentiated rate adaptation with a higher obtained QoE.

The remainder of this paper is organized as follows. In the next section, we provide a brief overview of related work in the area of HAS technology. Section 3 presents the proposed environment-aware QoE model, including the model establishment and verification. Section 4 introduces the environment-aware model-driven HAS rate adaptation algorithm. We then evaluate the performance of our proposal through experiments in Section 5. Finally, Section 6 concludes the paper and discusses the potential future works.

## 2. Related Work

### 2.1. QoE of Multimedia Service

The QoE of multimedia service is a subjective metric from the users’ perspective, which involves user expectations and perception of the multimedia contents. Many researches have been provided to understand, measure, and model QoE of multimedia services [5].

From the aspect of enhancing the user expectations of media service, a large amount of researches performs the video transmission with consideration of context and human factors. For example, many researches developed their QoE model based on physical, temporal, social, or even economic context. Some examples for the context are display screen, playing frequency, social scenario, and payment strategy [6]. For the human factor, researches [7,8] developed personalized multimedia content recommender and retrieve system for enhancing user experience.

On the other hand, from the aspect of improving user perception, QoE quality models are mainly established according to specific video streaming technologies, which concentrated on mapping the objective metrics to user perceptual quality. These QoE models are varying depending on the video streaming technologies and applications, such as QoE in social media [9], cloud gaming [10], and related HAS in this paper [2,3].

To sum up, a significant amount of researches is provided for the QoE management of multimedia service. Some of them have already considered the context-aware QoE models. However, the combination of QoE-driven HAS technology and context-aware HAS technology are still missing. We will further discuss them in the upcoming two subsections.

### 2.2. QoE-Driven HAS Technology

Currently, HAS technology has already been widely employed in commercial video streaming applications. In the academic domain, researchers are now more inclined to use QoE to evaluate adaptive strategies by optimizing the HAS algorithms based on the different QoE models. Most of the related studies on improving the QoE of HAS are client-driven approaches, they obey the client-centric nature of HAS [11,12,13,14]. Some researches have also focused on the server or network side [15,16,17]. These approaches try to find better alternative solutions for the management of concurrent requests, which deploy their solutions onto the servers, base stations, or software-defined networks.

For the client-driven approaches, the research [11] presented the results of the impact of resolution changes on user-perceived QoE in HAS and further demonstrated that the content type and resolution change patterns have significant impacts on the perception of resolution changes. The findings may help develop better QoE models and adaptation mechanisms. 360ProbDASH [12] designed a QoE-driven viewport adaptation system. It treats user’head movements as probability events and constructs a probabilistic model to depict the distribution of viewport prediction errors. A QoE-driven optimization framework is then proposed to minimize the total expected distortion of pre-fetched tiles. Besides, some research further focused on the machine learning approaches to maximize the QoE for end users [13,14]. For example, the research presented in Ref. [13] provided buffer-based reinforcement learning for HTTP adaptive streaming. The study proposed a reinforcement learning method to choose the segment quality during playback which uses only the buffer state information and optimizes this for a measure of user-perceived streaming quality. For the server or network side approaches, FINEAS [15] is a rate adaptation algorithm which is capable of increasing clients’QoE and achieving fairness in a multi-client setting. A key element of this approach is an in-network system of coordination proxies that are in charge of facilitating fair resource sharing among clients. Similarly, the research [16] also enforced video quality fairness among concurrent video flows generated by heterogeneous client devices in a software-defined networking (SDN) network. Furthermore, the research presented in Ref. [17] proposed an SDN-based dynamic resource allocation and management architecture for HAS systems. It allocates the network resources dynamically for each client based on their expected QoE and aims to alleviate these scalability issues and improve the per-client QoE.

To sum up, HAS have been intensively studied. QoE is becoming a significant indicator to evaluate the subjective perceptual quality of HAS services. However, the existing studies ignore the impact of the environment on the QoE evaluation of mobile users.

### 2.3. Context-Aware HAS Technology

Recently, some research has taken advantage of the available sensors or GPS information on mobile devices to enhance the performance of HAS. For example, Gtube [18] introduced an adaptive video streaming system for mobile users equipped with a GPS positioning sensor. The historical bandwidth information of the geographical location of the client is collected. In this manner, mobile users can send queries to the server in order to better predict the near-future bandwidth availability. GeoStream [19] also proposed a video streaming system that relies on the use of geostatistics to analyze the spatio-temporal bandwidth. The research focused on obtaining the accurate and predicted bandwidth values from unknown locations with a relatively sparse dataset. Similarly, MASERATI [20] also investigated the factors in natural environments and user contexts that affect the available link bandwidth. The research revealed that the humidity and location are the important factors in the 3G network, while the speed, time, and location are of great importance in the 4G LTE network when predicting the available link bandwidth. By using the information about the environments and contexts to predict the available bandwidth, MASERATI improves the performance of DASH in terms of the playout success rate, video quality, and stability. MDASH [21] proposed a mobility-aware, dynamic, adaptive streaming over HTTP to minimize the cellular data usage through a Markov Decision Process framework, which considers the mobile network types, bandwidth, last request bitrate, and the occupation of the buffer. EnvDASH [22] introduced an environment-aware adynamic adaptive streaming over an HTTP system. The system can detect the interest of a user watching a video stream, the instability of a device, and the ambient noise level, so as to reduce the generated network traffic.

To sum up, there are some context-aware HAS approaches that utilize the context information by sensors to promote their performance, such as location, the stability, and the network type. However, these approaches usually focus on the bandwidth prediction or network traffic reduction. The different criteria for evaluating video QoE under different environments of HAS remains intact.

## 3. Environment-Aware QoE Model for Video Streaming

The environment-aware QoE model is established by considering the differentiated effect of stalling and quality on QoE in different types of environments. Therefore, it is necessary to define and distinguish the different environments where the HAS mobile users are located. First, we use the sensor information of mobile phone to classify the environmental scenarios; Second, we analyze the effect of bit rate and stalling on QoE and then build the QoE model based on environment perception. Finally, we test and verify the proposed QoE model based on the MOS value and compare the result with the benchmark QoE model without considering the environment factors.

### 3.1. Sensor Data Collection from HAS Clients

This subsection first classifies the environment of HAS mobile users. We collect the sensor data of mobile users from a real mobile learning system as the training data set. The authors are in charge of developing the mobile learning system for the e-learning school of our university. For convenience, we record the sensor data of students, and further let students choose the reference environmental scenarios, such as the office, library, home, outdoor, car, or bus. In detail, the paper uses the sensor’s data from 871 Android users within half a year. The process of sensor data collection and saving is as follows. When the student starts to watch the video, the program starts a new thread to collect sensor data. The sensor type on the mobile client is obtained through the corresponding API in the Android SDK, and the sensor acquisition thread starts to monitor the sensor at the same time. The thread is acquired once every 0.05 s, that is, the acquisition frequency is 20 Hz. At the end of playback, the sensor data acquisition thread is immediately stopped and data is written to the file. Further, we also process the collected sensor data by low-pass filtering, high-pass filtering, and interpolation of missing values. Then, we select 28558 valid sensor data files accompanied by their environmental scenarios, making sure that each video viewing record is more than 10 min long. The data set is available and may be useful for other environment-aware studies [23].

### 3.2. Environment Classification

In order to establish an environment-based QoE model, we first make the following theoretical hypothesis, which is verified later in this paper. In a dynamic and noisy environment, the adaptive strategy of HAS should theoretically be more inclined to reduce the length of the stalling, so as to improve the fluency to enhance the user experience, while in a static and quiet environment, the HAS adaptive strategy should actively increase the average bit rate, thereby improving the user experience by improving the clarity.

Therefore, we first classify the environment into three categories: the dynamic and noisy environment, the static and quiet environment, and the intermediate state between the two environments. In order to distinguish between these three environments, in this study, we mainly focus on the triaxal acceleration and sound sensors of mobile clients.

**Figure 1:**
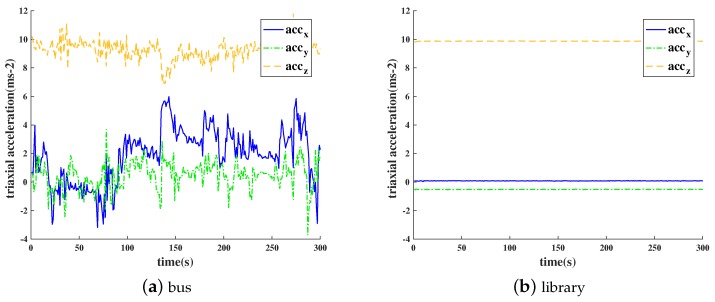
Acceleration sensor data over time in different environments. (**a**) Bus. (**b**) Library.

Through the analysis of the data set, we can distinguish the dynamic environment from the static scene only based on the value of the acceleration sensor. As shown in Figure 1, in the static scenes of libraries, the values of a triaxial acceleration sensor are basically stable, while in the dynamic scenes of buses, the sensor values change drastically. Therefore, the dynamic and static scenes can be distinguished by calculating the values of standard deviation of the triaxial acceleration sensor values.

Through a statistical analysis of sensor data from 400 randomly selected dynamic and static scenes, if the standard deviation of sensor data in any direction (x,y,z) of the triaxial acceleration sensor exceeds 3.0, we define this condition as dynamic. If the contrary occurs, we suppose that the condition is static. After the validation, the accuracy rate can reach 96% at the lowest.

**Figure 2:**
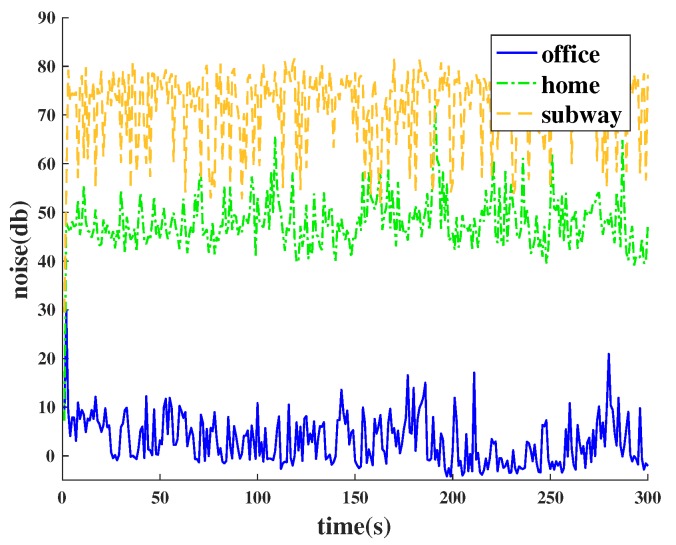
The noise data in different environments.

In the same way, we divide the noisy environment and quiet environment according to the average noise value of the microphone. According to the results of the statistical analysis shown in Figure 2, when the average noise exceeds 60 decibels, we define this condition as noisy; when the average noise is below 30 decibels, we think that this is a quiet environment. In our experiments, the accuracy rate can reach 97% at the lowest.

To sum up, we finally define and distinguish the three types of environment. The results are shown in Table 1, where *acc* represents the value of the standard deviation in the three directions of the accelerometer, and *voi* represents the mean value of the ambient noise in decibels.

In this manner, we define an environmental impact function *g* that is related to acc and voi. The function *g* can be used to construct an environment-aware QoE model.

### 3.3. Environment-Aware QoE Model

In the previous subsection, we have already defined three scenarios. In this subsection, we further present the scene-aware QoE model. We find that in the three different scenarios, the clarity and fluency have different impacts on the user’s perception of the video quality, thus realizing the environment-aware QoE model.

Taking into account the impacts of clarity, fluency, and the environment on QoE, the environment-aware QoE model can be constructed as follows:

(1) $$f=\left(1-\lambda \right)\bar{v}+g\left(acc,voi\right)\lambda \bar{s}$$

$\bar{v}$ and $\bar{s}$ respectively represent the influences of clarity and fluency on QoE. In this paper, their definitions are given by the following two formulas, which are derived from our benchmark scheme, BOLA [4]. BOLA is a buffer-based HAS scheme which has already been implemented in Dash.js [24]. Although BOLA does not define a unified QoE model, it does provide the metrics $\bar{v}$ and $\bar{s}$ about the clarity and fluency. Then, it formulates bitrate adaptation as a utility maximization problem, and devises an online control algorithm to minimize rebuffering and maximize video quality by stochastic optimization strategies. It should be noted that the definitions of $\bar{v}$ and $\bar{s}$ in the QoE model are not limited to their definitions in the BOLA algorithm.

g(acc,voi) is an environmental impact adjustment function. On the one hand, the function *g* can further balance the relationship between clarity and fluency. On the other hand, it makes different dimensions of $\bar{v}$ and $\bar{s}$ appear in the same formula. Finally, the value of λ represents the importance of fluency and clarity in the QoE evaluation and is influenced by many factors, for example, different video types, terminal types, etc. The method proposed in this paper is suitable for different λ values. λ values in this paper are not discussed (λ=0.5 by default).

Firstly, we give the definition of $\bar{v}$, which indicates the impact of clarity on QoE. As mentioned earlier, we adopt the concept of playback utility in BOLA to represent the contribution of clarity to user experience. We suppose that a video file can be divided into *n* segments, each of which has a length of *p* seconds, and each segment has *M* versions of the bit rate. According to the research presented in Ref. [25], there is a logarithmic relationship between the user experience and the average bit rate of the video. Therefore, for each version of the video segment, the logarithm of the video’s bit rate is used to represent the version, defined as $v_{m},m\in 1,2,...,M$, which is similar with the definition in BOLA. In this manner, $v_{m}$ can directly reflect the linear relationship between the bit rate and the user’s QoE.

Suppose that the timeline is divided into continuous and non-overlapping time slices, and the $k_{th}$ time slot starts at $t_{k}$, where k∈{1,2,...}. Then, the length of time slice $t_{k}$ can be denoted as $T_{k}=t_{k+1}-t_{k}$, which is a random variable that is determined by the actual network conditions. In HAS, at the beginning of each time slice $t_{k}$, the HAS rate adaptation algorithm decides whether to request a new video file. If the rate adaptation algorithm decides to download, the download length is $T_{k}$. Conversely, this time slice will have a fixed time interval of Δ. We adopt the variable $a_{m}$ in BOLA to indicate the video file’s request status at time $t_{k}$. In the light of the definition, $\sum_{m}a_{m}\left(t_{k}\right)\leq 1,\forall k$.

(2) $$a_{m}\left(t_{k}\right)=\left\{\begin{array}{cc} 1, & \mathrm{if}\;a\;\mathrm{segment}\;\mathrm{with}\;\mathrm{bit}\;\mathrm{rate}\;\mathrm{level}\\ & \mathrm{of}\;m\;\mathrm{is}\;\mathrm{requested}\\ 0, & \mathrm{if}\;\mathrm{no}\;\mathrm{segment}\;\mathrm{is}\;\mathrm{requested} \end{array}\right.$$

In this way, the contribution of the average bit rate or clarity to QoE can be described by the formula. In the formula, $T_{end}$ represents the time point when the viewing of video has finished.

(3) $$\bar{v}=\frac{E\{\sum_{k}\sum_{m}a_{m}\left(t_{k}\right)v_{m}\}}{E\{T_{end}\}}$$

Secondly, we give the definition of $\bar{s}$, which indicates the the impact of fluency on QoE, as shown in the formula.

(4) $$\bar{s}=\frac{E\{T_{end}-T_{stall}\}}{E\{T_{end}\}}$$

In this formula, $T_{stall}$ indicates the length of the stalling time.

To construct the environment-aware QoE model, we need to collect necessary information in Formula (1). First, ten volunteers in our lab are involved, who watch the video by phones in different environments and give MOS (Mean Opinion Score) results based on their feelings about clarity and fluency. Second, to ensure fairness, all volunteers watch the same video selected from the dash.js development community. The MPD (media presentation description) file can be found on the community website, which illustrates the parameters about the multiple versions of the video in detail [26]. In this manner, we have collected 96 valid MOS scores for the video viewing by filtering the raw data. These MOS scores are also used in the model validation in Section 3.4. Thirdly, we simultaneously collect the sensor data, the playback bit rates, and the length of stallings when volunteers are watching the videos. Then we work out the values of $\bar{v}$ and $\bar{s}$ respectively from the playback bit rates and the length of stallings.

**Figure 3:**
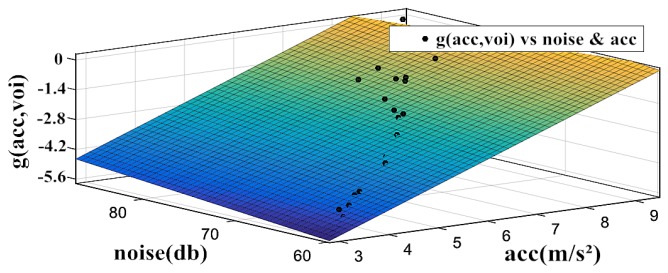
Curve fitting for the parameters in g(acc,voi).

Finally, the values of function g(acc,voi) are calculated by Equation 1 with the MOS scores of the video, $\bar{v}$, and $\bar{s}$. In this manner, we can draw the scatter plot with obtained *g(acc,voi)*, acc, and voi, as shown in Figure 3.

According to observations, we find that the g(acc,voi) conforms to the characteristics of linear functions with acc and voi under the context of $\bar{v}$ and $\bar{s}$. Therefore, according to Table 1, without losing the generality, g(acc,voi) is defined in Formula (5). It is obviously that other QoE models for clarity and fluency will result in different results of g(acc,voi), which may or may not conform to the characteristics of linear functions with acc and voi. Different target fitting function g(acc,voi) will be obtained in Formula (5). This ensures the generality of the proposed environment-aware QoE model.

(5) $$g\left(acc,voi\right)=\left\{\begin{array}{cc} \alpha (acc-3)+\beta (voi-60)+\gamma & \mathrm{acc}\;\geq \;3\;\&\;\mathrm{voi}\;\geq \;60\\ \alpha^{\prime }\frac{acc-3}{3}+\beta^{\prime }\frac{voi-30}{30}+\gamma^{\prime } & \mathrm{acc}\leq \;3\;\&\;\mathrm{voi}\;\leq \;30\\ 1 & \mathrm{others} \end{array}\right.$$

Consequently, we are able to use the curve fitting function to fit the *g* function by acc and voi in different environments, and calculate the parameters of g(acc,voi) in Formula (4). The parameter values in the environmental impact adjustment function under the context of BOLA are finally determined as follows in Formula (6) by curve fitting.

(6) $$g\left(acc,voi\right)=\left\{\begin{array}{cc} 1.97(acc-3)-0.2527(voi-60)-5.618 & \mathrm{acc}\;\geq \;3\;\&\;\mathrm{voi}\;\geq \;60\\ 0.072\frac{acc-3}{3}+0.248\frac{voi-30}{30}+1.34 & \mathrm{acc}\leq \;3\;\&\;\mathrm{voi}\;\leq \;30\\ 1 & \mathrm{others} \end{array}\right.$$

### 3.4. Model Validation

**Figure 4:**
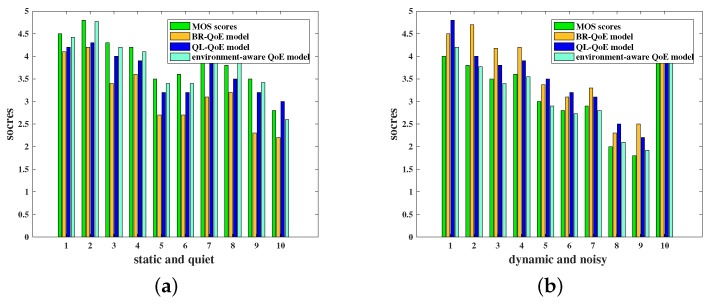
Ten groups of MOS score results in different environments. (**a**) Static and quiet environment. (**b**) Dynamic and noisy environment.

In order to verify the QoE model proposed in the above section, this paper also demonstrates the model through volunteer scoring. Volunteers watch videos of the same content in specific different environments with fluency and clarity as the main factors and scored the video according to their own subjective feelings. Then, the QoE values are also calculated based on the above environment-aware QoE model and two other QoE models named BR_QoE and QL_QoE without considering the environmental factors [27]. Figure 4 shows the QoE results for ten groups of the dynamic and noisy environment and the static and quiet environment, respectively.

For both the dynamic and noisy environment and the static and quiet environment, the value calculated by the environment-aware QoE model is closer to the real MOS scores than the models that does not consider the environmental factors. This demonstrates the correctness and validity of the environment-aware QoE model.

## 4. Environment-Aware HAS Rate Adaptation Algorithm

In this paper, the environment-aware HAS rate adaptation algorithm uses different rate adaptation strategies in different environments. If the environment-aware QoE model shows that the degree of fluency in the current environment contributes more to the user experience, it is necessary to actively avoid the occurrence of stalling when making rate adjustment decisions. If the model indicates that the degree of clarity in the current environment is more conducive to the user experience, it is necessary to actively improve the resource utility when making rate adjustment decisions. Without any loss in generality, we still select the formerly mentioned BOLA for the case study. It is taken as the benchmark for the environment-aware HAS rate adaptation algorithm to verify the effectiveness of our scheme. The BOLA algorithm belongs to a completely buffer-based rate adaptation algorithm. By analyzing the contribution of the average bit rate and the length of the stalling time to QoE, the algorithm obtains a near-optimal solution in each time slice for segment requesting. Since we adopt the definitions of playback utility and smoothness in BOLA when constructing the environment-aware QoE model, it was convenient for us to conduct a comparison study. It should be pointed out that the environment-aware HAS rate adaptation algorithm designed in this paper is applicable to all HAS adaptive strategies in which clarity and fluency could be balanced.

Therefore, this paper adjusts the BOLA algorithm by adjusting the objective function for each step with the the environmental impact adjustment function and designs the environment-aware BOLA algorithm based on the QoE model. We add the environmental impact adjustment function *g* to the BOLA. In the environment-aware BOLA algorithm, at the beginning of the $k_{th}$ time slice, if the formula $Vv_{m}+g\left(acc,voi\right)V\gamma p<Q\left(t_{k}\right)$ is satisfied for all bit rate levels, no new video block is requested in this time slice. Otherwise, it traverses the video segments at all bit rate levels and finds the targeting bit rate *m* that can maximize the value of the following equations. The detail explanation of the symbols is presented in Table 2.

(7) $$Maximize\frac{\sum_{m=1}^{M}a_{m}\left(t_{k}\right)\left(Vv_{m}+g\left(acc,voi\right)V\gamma p-Q\left(t_{k}\right)\right)}{\sum_{m=1}^{M}a_{m}\left(t_{k}\right)S_{m}}\;$$

(8) $$subject\;to\sum_{m=1}^{M}a_{m}\left(t_{k}\right)\leq 1.\;a_{m}\left(t_{k}\right)\in \left\{0,1\right\}$$

BOLA has demonstrated that it can achieve a near-optimal bit rate adaptation for the online videos with an optimal bit rate selection at each step. Correspondingly, the proposed environment-aware BOLA algorithm can also achieve a near-optimal bit rate adaptation for the user experience in varying environments. The optimal solution is to download the next chunk at bitrate index *m*, where *m* is the index of bit rate level that maximizes the ratio $\left(Vv_{m}+g\left(acc,voi\right)V\gamma p-Q\right)/S_{m}$ among all *m* for which this ratio is positive.

In this manner, when the acceleration value of the client is greater than 3.0 and the ambient noise is greater than 60 dB, that is, in a dynamic and noisy environment, it guarantees higher fluency and QoE. When the acceleration value of the client is less than 3.0 and the ambient noise is lower than 30 dB, that is, in a static and quiet environment, the average bit rate increases, and at the same time, the influence of fluency on QoE is reduced, which in turn improves QoE.

## 5. Experimental Evaluation

### 5.1. Experiment Setup

In this section, we discuss the implementation of the proposed environment-aware HAS rate adaptation scheme and its benchmark in a DASH (Dynamic Adaptive Streaming over HTTP) streaming system to demonstrate the efficiency of the proposed scheme in a real-world experimental environment. For the server side, we used the Apache server (version 2.2) deployed on the public Internet to handle HTTP requests. The Apache server is in charge of responding to the clients’ requests. For the HAS client, we integrated a DASH player named dash.js [24] in a self-developed android client. Firstly, according to the proposed method, BOLA is rebuilt into an environment-aware BOLA algorithm, noting that the BOLA algorithm has already been implemented in dash.js. Secondly, the sensor acquisition function is integrated into the client. Thirdly, a scoring module is implemented for the QoE evaluation by volunteers. Using the scoring module, 381 valid MOS scores about the environment-aware BOLA and its benchmark are obtained by the ten volunteers. The watched video is the same one for QoE modeling. Finally, the experimental environments of this algorithm include the campus, bus, home, library, and so on.

### 5.2. Experimental Results

The metrics to evaluate the effectiveness of the proposal are the average bit rate, the stalling time, and the QoE scores from both the model calculation and the volunteer evaluation. In the experiment, the average bit rate levels and stallings are collected when the volunteers watch the specified video.

**Figure 5:**
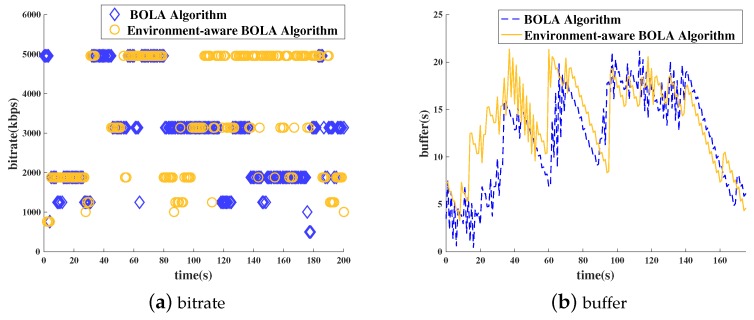
Bitrate and buffer (quiet and static environment): (**a**) bitrate, (**b**) buffer status.

Figure 5 shows the results of experiments in static and quiet environments, such as in the library or home. Figure 5a shows the results of the requested bit rate in these environments. It can be clearly seen that the environment-aware BOLA algorithm is more aggressive at the requested bit rate level. Figure 5b shows the buffer status in quiet and static environments. As can be seen from the figure, neither of the two algorithms in the two environments had a stall, indicating that the network is in a good and stable condition in this environment. From the above results, it is clear that the context-aware BOLA algorithm increases the clarity by increasing the requested bit rate in a static and quiet environment. At the same time, there is no major changes in the probability of stallings.

**Figure 6:**
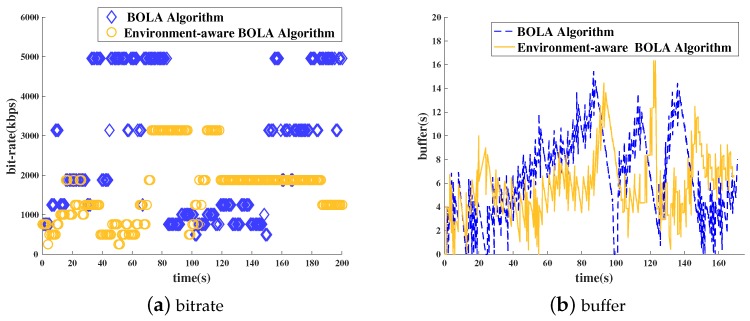
Bit rate and buffer (dynamic and noisy environment).

Figure 6 shows the experimental results when the user is sitting on the bus or walking on campus. These environments are basically dynamic and noisy. Figure 6a shows the results of the requested bit rate in these environments. It can be seen that the environment-aware BOLA algorithm is more gentle at the requested bit rate level. In this time, the bit rate levels is lower than the BOLA algorithm. However, from the results in Figure 6b, it is clear that the length of stallings is reduced obviously.

Table 3 shows the bit rate, stalling times results in detail. Furthermore, it presents two kinds of QoE scores. On the one hand, we collect the MOS scores from the volunteers who participate in the evaluation. On the other hand, the QoE values are calculated by the environment-aware QoE model.

According to the experimental results, in a static and quiet environment, compared with the original BOLA algorithm, the environment-aware BOLA algorithm can maintain an approximation in the stalling result, but it can significantly increase the requested average bit rate, thereby increasing the clarity of the video. In the dynamic and noisy environment, compared with the original BOLA algorithm, the environment-aware BOLA algorithm can significantly reduce the number and time of occurrence of stallings while somehow reducing the average bit rate. Since the bit rate and stalling weigh differently in these two environmental scenarios, all of the above results will lead to higher QoE. As shown in the table, if we bring experimental data into the environment-aware QoE model, the QoE value based on the environment-aware BOLA optimization algorithm is significantly better. This result is expected, because we use the QoE model as the optimization goal at each step. The MOS results further demonstrate the efficiency of the proposal.

## 6. Conclusions

This paper proposes an environment-aware HAS scheme for the differentiated QoE evaluation criteria in different environments. The scheme first establishes and quantifies an environment-aware QoE model to distinguish the importance of the clarity and fluency to QoE evaluation in different environments. Based on the QoE model, an environment-aware HAS rate adaptation algorithm is further proposed. In various environments, utilizing the sensor data in mobile phone, the value of an environment adjustment function can be calculated, which optimizes HAS strategies in different environments. We evaluate the scheme in real mobile environments. Experimental results show that the proposed scheme adopts a differentiated bit rate adaptation in different environments and ensures that an optimized QoE result can be obtained.

In this study, we propose an environment-aware HAS method. This method mainly considers the two-dimensional factors of the environment, whether it is moving or stationary and quiet or noisy. However, there are many other types of context information related to mobile users, such as the device type, network type, and user behaviors. Therefore, in future work, we will further explore the relationship between various types of context information and the QoE evaluation methods. In particular, we will further improve and enrich the environment-aware QoE model in actual video-on-demand applications, so as to provide video services with a higher QoE.

## Figures and Tables

**Table 1:** Environment classification.

Acc and Voice	Environment
acc≥3&voi≥60	dynamic and noisy
acc≤3&voi≤30	quiet and static
others	normal

**Table 2:** Explanation of symbols.

Symbol	Description
$t_{k}$	$k_{th}$ time segment
m	bit-rate level 1 to m
$a_{m}\left(t_{k}\right)$	request m-level video segment in $k_{th}$ time
$S_{m}$	the size of the m-level video segment
p	the length of the video
$v_{m}$	the contribution to QoE (bit-rate)
V	controlling parameter
γ	the contribution to QoE (stalling)
$Q(t_{k})$	the buffer space of $k_{th}$ time
g(acc,voi)	the environmental impact adjustment function

**Table 3:** Detailed experimental results in different environments (bolded values show the better results).

Environment	Algorithm	Bit Rate	Stallings	*g*-QoE(*f*)	MOS
static and quiet	BOLA	3639.51	0	3.61	3.79
	environment-aware BOLA	**4335.46**	0	**4.25**	**4.05**
dynamic and noisy	BOLA	**3553.32**	13	2.97	3.09
	environment-aware BOLA	2551.91	**4**	**3.92**	**3.79**
